# Heterogeneity of clinical features, EEG and brain imaging findings in anti-leucine-rich glioma-inactivated protein 1 autoimmune encephalitis: a retrospective case series study and review of the literature

**DOI:** 10.1186/s42494-023-00132-5

**Published:** 2023-08-15

**Authors:** Emily Yixuan Huang, Hongfeng Gao, Ning Zhong

**Affiliations:** 1 Department of Molecular and Cell Biology, University of California, Berkeley, CA 94720 USA; 2 Department of Molecular and Cell Biology and Helen Wills Neuroscience Institute, University of California, Berkeley, CA 94720 USA; 3 Department of Neurology, Kaiser Permanente Sacramento Medical Center, Sacramento, CA 95825 USA; 4 North Valley Regional Epilepsy Center, Kaiser Permanente Sacramento Medical Center, Sacramento, CA 95825 USA

**Keywords:** Anti-leucine-rich glioma-inactivated 1, Autoimmune encephalitis, Faciobrachial dystonic seizures, Epilepsy

## Abstract

**Background:**

Anti-leucine-rich glioma-inactivated 1 (LGI-1) autoimmune encephalitis (AE), characterized by rapid decline of memory, seizures, and neuropsychiatric abnormalities, is a rare but devastating disorder. Early diagnosis and treatment are essential to prevent long-term sequelae. In this report, we provide a detailed description of clinical characteristics, laboratory test results, imaging, and electroencephalography (EEG) findings, as well as treatment responses of eight patients with anti-LGI-1 AE treated at our center.

**Case presentation:**

At the onset, all eight patients presented with confusion/memory deterioration, seizures (including faciobrachial dystonic seizures or other types of seizure), and behavioral changes such as hallucination, paranoia, and anxiety. Four patients were found with severe hyponatremia. Anti-LGI1 antibodies were detected in the cerebrospinal fluid and/or serum of all patients. For patients with faciobrachial dystonic seizures, no discernible scalp EEG change was detected, while EEG recording of patients experiencing other types of seizure showed focal slowing, focal epileptiform discharges, and focal onset seizures. All patients showed abnormal brain magnetic resonance imaging signals, mainly involving the mesial temporal lobe and the hippocampus. In addition, one patient also experienced fulminant cerebral edema during the acute phase of the illness. All patients received immunotherapy and anti-seizure medications and achieved good seizure control. Nevertheless, these patients continued to experience cognitive impairment during their long-term follow-ups.

**Conclusions:**

The care of anti-LGI1 AE patients requires rapid evaluation, prompt initiation of immunotherapy, and long-term follow-up. The long-term presence of neurocognitive complications observed in these patients underline the importance of developing reliable biomarkers that can distinguish between different subtypes of this disease with heterogeneous clinico-electrographico-radiological features. Further research is needed to understand the molecular mechanisms underlying the heterogeneity, in order to facilitate development of more effective treatments for anti-LGI1 AE.

**Supplementary Information:**

The online version contains supplementary material available at 10.1186/s42494-023-00132-5.

## Background

It has been over two decades since the discovery of voltage-gated potassium channel (VGKC) complex-related autoimmunity. The VGKC-complex antibodies do not directly interact with the VGKC complexes themselves; instead, they bind to two closely associated proteins: leucine-rich glioma-inactivated 1 (LGI1) and contactin-associated protein-like 2 (Caspr2) [1]. Since the pioneering work by Dalmau's group in 2010, anti-LGI1 autoimmune encephalitis (AE) has been recognized as the most common cause of limbic encephalitis and the second most common cause of AE [2, 3]. Diagnosis of anti-LGI1 AE is based on the detection of anti-LGI1 antibodies in the serum and/or the cerebrospinal fluid (CSF). Anti-LGI1 AE is a heterogeneous condition, as patients typically experience limbic symptoms such as rapidly declining memory and neuropsychiatric/behavioral problems; faciobrachial dystonic seizures (FBDS) or other types of seizure; and metabolic derangements such as hyponatremia [4]. The occurrence of anti-LGI1 AE may vary depending on age and sex, with males over the age of 60 being the most affected group. In some patients, the anti-LGI1 AE may be caused by an underlying tumor or other medical conditions that may trigger the autoimmune response, while others may have no identifiable causes. The heterogeneity of this condition highlights the importance of individualized examination. Timely detection of anti-LGI-1 antibody in either CSF and/or the serum and prompt use of immunotherapy are crucial for a favorable treatment outcome. The clinical course of anti-LGI1 AE is complex, but patients typically respond well to immunotherapy [5].

In this report, we present eight patients diagnosed with anti-LGI1 AE, in the aim to help clinicians better recognize, diagnose, and treat this disease.

## Case presentation

We retrospectively analyzed the clinical data, electroencephalography (EEG) and imaging findings, treatment, and outcomes of eight patients diagnosed with anti-LGI1 AE at the Neurology Department of Kaiser Permanente Sacramento Medical Center between 2015 and 2022. The patients included five females and three males who met the following inclusion criteria: (1) rapid onset (within 3 months) of symptoms of memory impairment and/or mental and behavioral abnormalities; (2) occurrence of seizures during the acute or subacute phase of the disease; (3) detection of anti-LGI1 autoantibody in the CSF and/or the serum.

### Patients

The patients’ mean age at symptom onset was 64 ± 11 years (median 63.5 years). The latency from the onset of clinical symptoms to confirmed diagnosis ranged from 0.6 to 5 months (mean 2.4 ± 1.6 months, median 2 months), and the follow-up period ranged from 10 to 72 months. Table 1 summarizes the patients' clinical data.

**Table 1:** Clinical manifestations of the patients

			Case #1	Case #2	Case #3	Case #4	Case #5	Case #6	Case #7	Case #8
Gender			Female	Male	Female	Female	Female	Male	Female	Male
Age at onset (years)			49	79	63	57	79	64	58	60
Latency to diagnosis (months)			2	4	4.5	2	0.6	0.6	4	1
Symptoms	Initial symptoms		Confusion, short-term memory difficulty, speech difficulty, seizures	Decline of short-term memory difficulty, seizures	Confusion, poor memory, seizure	Confusion, seizure	Confusion, speech difficulty, rapid deterioration, obtunded	Confusion, seizure	Seizure, short-term memory difficulty	Confusion, short-term memory difficulty, personality changes, seizures
	Psychiatric symptoms		Hallucination, paranoia, anxiety	Anxiety	Anxiety, paranoia	Paranoia, anxiety	Hypersomnia	Confusion, hallucination	Hallucination, anxiety, agitation	Confusion, talkative, personality changes
	Seizure semiology		Focal dyscognitive seizures	FBDS	FBDS, focal dyscognitive seizures	FBDS	No clinical seizure at onset	Focal dyscognitive seizures, FBDS	Focal dyscognitive seizures	Focal dyscognitive seizures
Pertinent lab study results	LGi1 antibody (serum)		Not tested	+1:1280	Not tested	+	+	+ 1:640	+	+
	LGi1 antibody (CSF)		+	+	+	+	+	ꟷ	+	+
	Other autoantibodies		AchR antibodies and striated muscle antibodies in serum	Neuronal VGKC in serum	Anti-AchR Ganglionic, anti-TPO in serum	Neuronal VGKC in serum	Neuronal VGKC in serum	GAD65 (low titer), Neuronal VGKC in serum	None	None
	Serum sodium (normal 135–155 mmol/L)		120	136	118	121	136	117	136	133
	CSF Results	WBC count (CSF) (normal 0–5/µL)	4	19	8	3	6	2	3	1
		Protein (CSF) (normal 15–45 mg/dL)	46	102	49	22	349	36	32	46
		OligoClonal Band (CSF)	Abnormal, type 3	Normal	Abnormal, type 3	–-	–-	–-	Normal	Abnormal, type 2
Brain Imaging	Initial results		FLAIR/T2 hyperintensity seen in the bilateral mesial temporal lobes	No specific findings in MRI, PET showed hypometabolism in bilateral posterior cingulate gyrus and precuneus	Unremarkable	Unremarkable	Diffuse cortical edema, swelling throughout both cerebral hemispheres, left more than right	FLAIR/T2 hyperintensity seen in the left mesial temporal lobes	Unremarkable	Unremarkable
	Follow up results		Gliosis noted in mesial temporal lobe	Brain atrophy noted	Increased FLAIR/T2 signal in the mesial temporal lobes bilaterally, mild right hippocampal atrophy	Signal changes in the left anterior-medial temporal lobe	Bilateral diffuse cerebral edema, worse in the left temporal regions	Mild left hippocampal atrophy	Hyperintense FLAIR/T2 signal within the bilateral mesial temporal lobes	Increased FLAIR/T2 signal in the mesial temporal lobes bilaterally, mild hippocampal atrophy
EEG	Interictal		Rare epileptiform discharges in the left temporal, focal slowing seen in either temporal region	Normal	Focal slowing in the left temporal regions	Normal	Diffuse slow, lateralized periodic discharges in the left hemisphere	Diffuse slow background recording	Focal slowing in the left temporal regions, L TIRDA	Normal
	Ictal (vEEG)		–-	No cerebral EEG changes at clinical events	Focal-onset seizures arising from the left temporal regions	Focal slowing in the left temporal regions, no cerebral EEG changes at clinical events	Non-convulsive status	Focal-onset seizures arising from the left fronto-temporal regions	Focal-onset seizures arising from the left temporal regions	Focal-onset seizures arising from the right temporal regions
Tumor identified			Thymoma	None	None	None	None	None	None	None
ASM			Levetiracetam, Lacosamide	Levetiracetam, carbamazepine	Levetiracetam, phenytoin	Levetiracetam, phenytoin	Levetiracetam, valproic acid, Lacosamide, phenytoin	Levetiracetam, Lacosamide	Levetiracetam	Valproic acid, clobazam
Immunotherapy		First line	Methylprednisolone, IVIG, PLEX	IVIG	Methylprednisolone, IVIG	Methylprednisolone, IVIG, PLEX	Methylprednisolone, IVIG	IVIG, Methylprednisolone	Methylprednisolone	Methylprednisolone, IVIG
		Second line	Rituximab, Cyclophosphamide	None	None	Rituximab	None	Rituximab	None	Rituximab
Time to Immunotherapy (days)			30	120	150	40	20	60	100	30
Follow up	Follow up duration (months)		30	46	60	72	12	10	10	10
	Relapse (months)		Yes (3 months)	None	Yes (4 months)	Yes (8 months)	Yes (2 months)	Yes (3 months)	None	Yes (1 month)
	Cognitive assessment^a^		Major neurocognitive disorder with deficits in declarative memory and executive function, and persistent poor verbal memory	MOCA 20/30 MMSE 23/30 Mild neurocognitive disorder, Difficulty with delayed recall and visual executive function	Mild neurocognitive disorder, Difficulty with phonemic verbal fluency and impaired graphomotor construction	Moderate neurocognitive disorder, Difficulty with visuospatial abilities, short-term memory, and executive systems function	Severe neurocognitive disorder, MOCA: 0/30 Continued with poor memory; intermittent confusion and in need of maximal assistance for daily physical activities	Mild to moderate neurocognitive disorder tested by CLQT, Difficulty with short-term memory and visual executive function, Intermittent confusion	Moderate neurocognitive disorder tested by CLQT, Difficulty with short-term memory and visual executive function	Moderate to severe neurocognitive disorder, MOCA 9/30, Difficulty with visual executive function, language, memory, and attention, Intermittent confusion
	Functionality assessment^b^- mRS	Onset	5	1	5	3	5	5	4	5
		Last follow-up	3	1	3	2	4	2	2	3
		∆mRS	2	0	2	1	1	3	2	2
	Seizure		No (ASM weaning)	No (ASM weaning)	Continued with refractory focal dyscognitive seizures with reduced frequency	No (ASM weaning)	No (still taking ASMs)	No (still taking ASMs)	No (still taking ASMs)	No (still taking ASMs)

*FBDS* Faciobrachial dystonic seizures, *AchR* Acetylcholine receptor, *VGKC* Voltage-gated potassium channel, *TPO* Thyroid peroxidase, *GAD65* Glutamic acid decarboxylase 65-kilodalton isoform, *IVIG* Intravenous immune globulin, *PLEX* Plasmapheresis, *CLQT* Cognitive Linguistic Quick Test, *ASMs* Anti-seizure medications, *mRS* Modified Rankin Scale

^a^Comprehensive neuropsychology tests were obtained for cases #1, #3, #4; the following test batteries were used: Beck Depression Inventory (BDI-II); Boston Naming Test; Burns Anxiety Inventory; California Verbal Learning Test-Third Edition (CVLT-3 Short form); Controlled Oral Word Association (FAS, Animal Naming); Delis Kaplan Executive Systems Function (D-KEFS; select subtests); Hooper Visual Organization Test; Neuropsychological Assessment Battery- Memory, Language, and Screening Modules (NAB; select subtests); Rey Complex Figure Test; Trail Making Test: Parts A & B; Wechsler Memory Scales-Fourth Edition (WMS IV, selected subtests); Wechsler Adult Intelligence Scale, Fourth Edition (WAIS IV, select subtests); Wide Range of Achievement Test, Fifth Edition (WRAT-5, word reading test); and Wisconsin Card Sorting Test

^b^mRS was assessed at the onset of symptoms during hospitalization and at last clinic follow-up; ∆mRS represents the score at hospitalization minus that at the last clinic visit

### Clinical features

The primary symptoms at onset were seizures and cognitive disorders. In addition to the most obvious cognitive disorders (memory impairment and intermittent confusion with acute or subacute onset), the patients also presented with other neurobehavioral/psychiatric symptoms, including paranoia and anxiety (*n* = 5 patients), personality changes (*n* = 1), hallucinations (*n* = 1), and hypersomnia (*n* = 1). These neurocognitive/neuropsychiatric manifestations led to earlier suspicion of autoimmune etiology in five patients, and AE panel testing was requested. Serum studies revealed severe hyponatremia in four patients. The CSF profile was less impressive, with only samples from two patients showing signs of pleocytosis. Five patients showed elevated CSF protein levels. Abnormal oligoclonal bands were seen in three of five patients tested (Table 1). Other autoantibodies were also detected in sera of 6 patients (Table 1). Tumor/cancer screening led to the discovery of thymoma in one patient (case #1) who was tested positive for anti-acetylcholine receptor antibodies and striated muscle antibodies in the serum. Case #1 did not have clinical symptoms of myasthenia gravis, nor did the neurodiagnostic testing with nerve conduction studies and electromyography reveal any evidence for myasthenia gravis.

Seizures were manifested as FBDS in four patients and as focal-onset seizures with impaired awareness (focal-onset dyscognitive seizures or complex partial seizures) in five patients. One patient did not exhibit clinical seizures at onset, but EEG monitoring confirmed non-convulsive status epilepticus. Three patients (cases #2, #3, and #4) presented with FBDS at onset. They were misrecognized as focal motor seizures or myoclonus seizures initially, and FBDS were not suspected until the patients were referred to a tertiary epilepsy center and underwent video EEG monitoring (vEEG). Thus, the failure to recognize FBDS caused a delay in making the accurate diagnosis. Once vEEG monitoring confirmed the diagnosis of FBDS, further tests were prompted for these patients, and the test results confirmed the diagnosis; therefore immunotherapies were initiated. In our series of cases, we initiated immunotherapies after the positive antibody diagnosis was confirmed for case #2, case #3, and case #6. Immunotherapies were initiated while waiting for confirmation of diagnosis for case #4 (Table 1).

### EEG findings

**Fig. 1:**
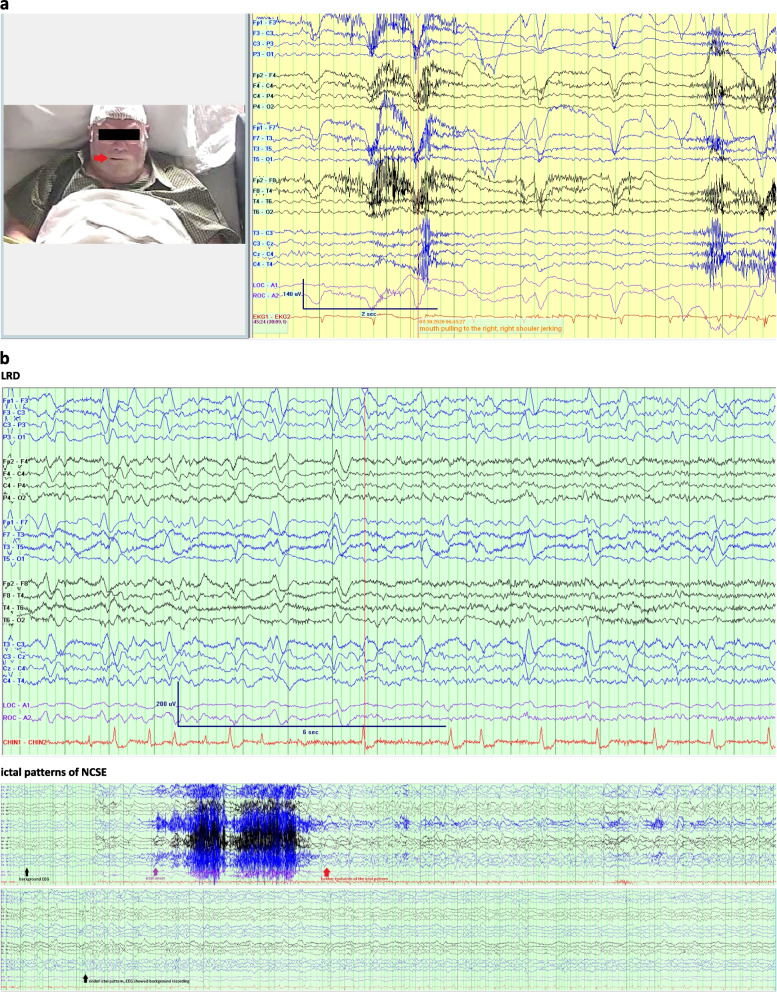
**a** During faciobrachial dystonic seizure (FBDS), EEG recording did not show discernible changes when the patient displayed mouth tonic twitching (as indicated by the red arrow) (case #2).** b** Lateralized rhythmic discharges (LRD, prominently seen in the left hemisphere) were seen in the case #5 with non-convulsive status epilepticus (NCSE, ictal patterns recognized as asynchronized bilateral rhythmic triphasic-morphology sharp discharges with discernible evolutions with frequency and voltage amplitude) and fulminant cerebral edema

Notably, FBDS were not accompanied by discernible cerebral EEG pattern changes during clinical events as recorded by vEEG monitoring (Fig. 1a). The most common EEG findings in our patients were (1) interictal focal slowing in the temporal regions, and (2) focal-onset seizures arising from the temporal regions when ictal patterns were recorded (Table 1). Lateralized rhythmic discharges (LRDs) in the left fronto-temporal regions (Fig. 1b) were observed in a patient who later developed non-convulsive status, presenting with asynchronized bilateral rhythmic triphasic-morphology sharp discharges with discernible evolutions (Fig. 1b).

### Imaging results

**Fig. 2:**
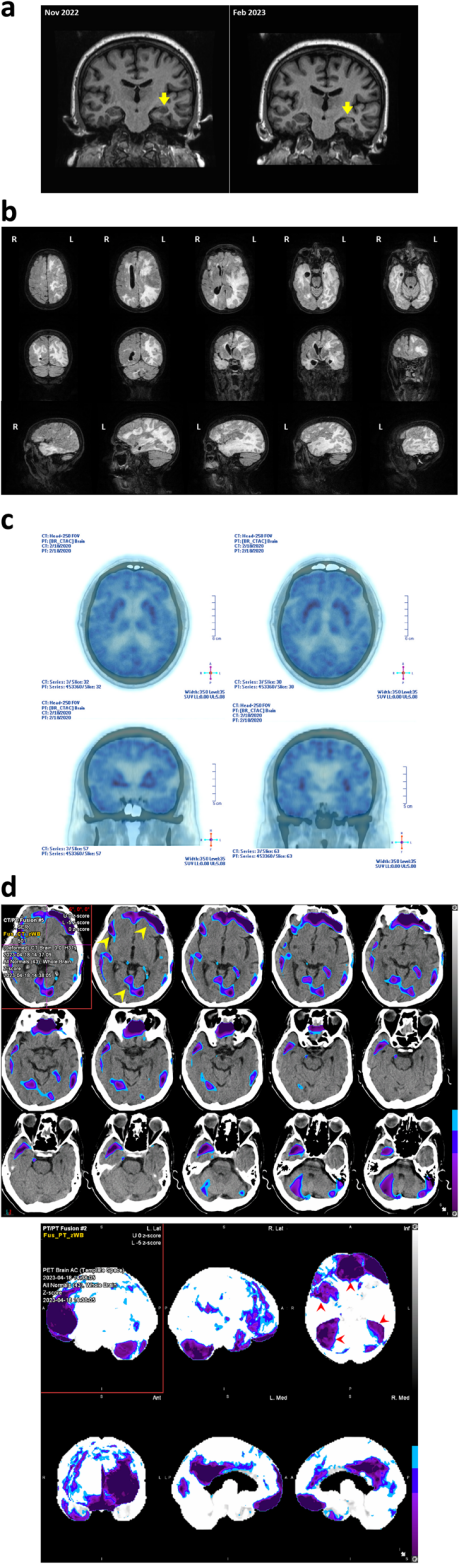
**a** The case #6 developed asymmetric hippocampal atrophy as seen in serial brain MRI images. **b** Fulminant cerebral edema seen in case #5, more in the left hemisphere. **c** Brain PET revealed hypermetabolism in the basal ganglion in case #2 with FBDS. **d** The case #8 showed bilateral hypometabolism in the frontal and temporal lobes with rapid neurocognitive decline (brain PDG-PET scan); biodistribution shows statistically significant hypometabolism with very low Z-scores (-3, shown as purple-blue pseudo-color) in the posterior cingulate gyrus, as well as frontal (left worse than right) and temporal lobes (right worse than left). Upper panel shows the spatial structural localization of the hypometabolism; lower panel shows the fusion of images with low structural resolution to highlight the affected brain regions

All patients showed abnormal signals in serial brain magnetic resonance imaging (MRI) examinations. Signal changes in the mesial temporal lobe were observed in all patients (Table 1 and Fig. 2a). In one patient (case #5), brain MRI showed diffuse cortical edema that progressed from supratentorial to subtentorial regions within 10 days, representing fulminant cerebral edema (Fig. 2b). Brain FDG-PET (fluorodeoxyglucose positron emission tomography) was performed in one patient with significant FBDS (case #2), revealing hypermetabolism in bilateral basal ganglia (Fig. 2c). In another patient (case #8), brain FDG-PET at 3 months after disease onset showed significant bilateral hypometabolism in the frontal lobes, temporal lobes, and posterior cingulate gyri (Fig. 2d).

### Treatment and outcomes

All patients received anti-seizure medications (ASMs) as the first-line treatment. Two patients were empirically treated with antiviral medications at the onset of symptoms, and three patients received prompt immunotherapies while the autoimmune panel results were still pending. The average delay of immunotherapy initiation was 2.4 ± 1.6 months (median, 2 months) after symptom onset. Intravenous infusion of methylprednisolone and intravenous immune globulin were administered to all patients, and one patient also received plasma exchange therapy. Three patients received second-line immunotherapies with rituximab (Table 1). The patients experienced notable improvement of FBDS symptoms and other types of seizure after initiation of the immunotherapies. Only one patient suffered refractory focal dyscognitive seizures and required escalation of ASMs. None of the other patients experienced any recurrent seizures during the follow-up, and three of them successfully achieved ASM weaning.

During the 10–72 months of follow-up, our patients showed notable improvement of seizures and neurobehavioral/psychiatric symptoms. However, all patients continued to experience cognitive difficulties ranging from mild executive functioning, poor short-term memory to more severe neurocognitive disorders (Table 1), which were likely to affect their functional status measured by modified Rankin Scale (mRS). Six patients experienced at least one relapse with neurocognitive symptoms such as confusion or changes in the consciousness level, requiring hospitalization and escalated immunotherapies. The patient with fulminant cerebral edema continued to suffer from intermittent confusion and remained bed-bound during 3–6 months of follow-up. Maximal assistance was provided to this patient for daily activities such as feeding, ambulation, and transfer from bed to commode. At 12 months of follow-up, the patient showed some improvement in physical activity but remained with cognitive difficulty (Table 1).

## Discussion

With the discovery of autoimmune encephalitis and the identification of autoantibodies targeting cell surface or synaptic proteins, the number of reported anti-LGI1 AE cases has increased exponentially. However, it remains challenging for healthcare providers to recognize and promptly treat patients with this type of AE due to the heterogeneity of clinical manifestations and the dependence on detecting neuronal autoantibodies [6]. Anti-LGI1 AE now accounts for 11.2% of all autoimmune encephalitis cases, the latter of which is also the most common form of limbic encephalitis. The annual incidence of anti-LGI1 encephalitis is estimated to be 0.83–2 per million persons [7]. Although the exact number of reported cases of LGI1 encephalitis is unclear, a recent systemic review has identified approximately 500 cases reported in literature [8]. Seizures, cognitive impairments, hyponatremia, and abnormal brain MRI T2/FLAIR signals are among the mostly observed clinical manifestations in patients with anti-LGI1 AE [4]. Unlike anti-NMDA AE, which presents more characteristic chronological clinical phases, patients with anti-LGI1 AE present more heterogeneous clinical features as observed in our cases [9]. Such heterogeneity in clinical features can make early and precise diagnosis of anti-LGI1 AE even more challenging.

### Heterogeneity of seizure presentation

Patients with anti-LGI1 AE can present different types of seizure in addition to FBDS. In a recent study, patients were subgrouped based on seizure semiology: FBDS alone (FBDS), epileptic seizures without FBDS (non-FBDS), and coexistence of FBDS and other seizures (FBDS +) [10]. FBDS is the most common characteristic semiology, involving ipsilateral (less frequently, bilateral) dystonia-like seizures involving the face and/or the limb. It is estimated that 20-40% of the patients with anti-LGI1 AE have FBDS. However, FBDS is often misrecognized as focal motor or focal myoclonic seizures in general neurology practice due to its relative rarity. In addition, scalp EEG is often negative during FBDS, and the lack of ictal EEG pattern changes makes diagnosis challenging, leading to treatment delays [11]. In our case series, FBDS was not recognized in three patients until their referral to specialized epilepsy programs, representing challenges in diagnosis that often lead to treatment delays. One possible explanation for why scalp EEG fails to detect ictal pattern changes during FBDS is that FBDS is correlated with basal ganglia pathology, of which the electrophysiology changes are often difficult to be detected by scalp EEG [11]. In our patients, focal dyscognitive seizures (focal onset seizures with impaired awareness) and FBDS were two main types of seizure, which may correspond to hippocampus (mesial temporal types of seizures) and/or basal ganglia, two main targets of anti-LGI1 autoantibodies [11]. During treatments for seizure control, FBDS usually show a robust response to immunotherapies compared to other clinical symptoms. The presence of different types of seizures in addition to FBDS may indicate the involvement of a multi-foci epilepsy network, which may account for different seizure semiology and imply a poor prognosis [11]. Most of our patients showed promising seizure control outcome, and three patients even successfully achieved ASM weaning. Only one patient suffered from drug-resistant epilepsy, which might be correlated to the delay in diagnosis and initiation of immunotherapies.

### Heterogeneity of brain imaging findings

At the acute phase of anti-LGI1 AE, cerebral involvement is rare. In this report, one of our patients had fulminant cerebral edema, a severe and rapidly progressing form of cerebral pathology often observed in patients with infectious encephalitis but rarely in patients with AE. Other etiologies of the fulminant cerebral edema such as infectious meningoencephalitis, cerebral angiitis, intoxication, and primary central nervous system (CNS) malignancy were ruled out (see Additional file 1: supplemental data). Significant elevation of CSF proteins indicated severe CNS inflammation secondary to the immune response induced by anti-LGI1 autoantibodies, which may potentially interrupt the blood–brain barrier integrity and lead to extensive cerebral inflammation. To our knowledge, this is the first report of fulminant cerebral edema in anti-LGI1 AE. For this patient, recognizing a possible autoimmune etiology prompted medical intervention, which prevented potentially life-threatening detrimental complications.

Abnormal T2/FLAIR signals in the mesial temporal structures are commonly observed in anti-LGI1 AE patients and are believed to be associated with cognitive symptoms [12]. Neuroimaging studies have identified structural and functional differences between anti-LGI1 AE patients and healthy individuals, revealing that the hippocampus, amygdala, and other mesial temporal structures are the most affected brain structures [12]. In the acute phase, the most characteristic alterations are edema and T2/FLAIR hyperintensity in the hippocampus and the mesial temporal structures. Additionally, extratemporal lesions such as basal ganglia hyperintensities have been reported in patients with FBDS during the acute phase [13]. Beyond the subacute phase, the degree of hippocampal atrophy is associated with the severity of long-term cognitive impairment [14]. In our case series, we observed hippocampal atrophy in 4 patients and mesial temporal sclerosis in one patient. The mesial temporal and hippocampal atrophy persisted even after disease remission. In one patient, the brain FDG-PET showed hypometabolism patterns similar to those with fronto-temporal lobe dementia at as early as 3 months after disease onset. These findings indicate that with disease progression of anti-LGI 1 AE, critical structures essential for learning, memory, and executive function are rapidly damaged, leading to long-term or permanent hippocampal and global cerebral dysfunctions despite receiving immunotherapies [15–17]. In this report, most of the patients had positive outcomes during long-term follow-up after seizure treatment, yet persistent deficits in memory were observed. This highlights the critical importance of early and effective immunotherapy [16]. In our case series, three patients were only treated with ASMs when presenting with FBDS or other types of seizure. Two patients (cases #6 and #8) experienced relapse with profound neurocognitive impairment. After consultation with a specialized epilepsy program, immunotherapy was initiated, and the patients achieved good neurobehavioral recovery. Unfortunately, brain MRI showed notable hippocampal atrophy, which was likely associated with the lingering poor short-term memory of these patients (Fig. 2a). Other published case studies have shown that FBDS often precede cognitive decline. Therefore, recognizing FBDS before other clinical manifestations and treatment with immunotherapy may help reduce the long-term complications.

### Molecular mechanism of the anti-LGI1 antibody-induced pathogenesis

**Fig. 3:**
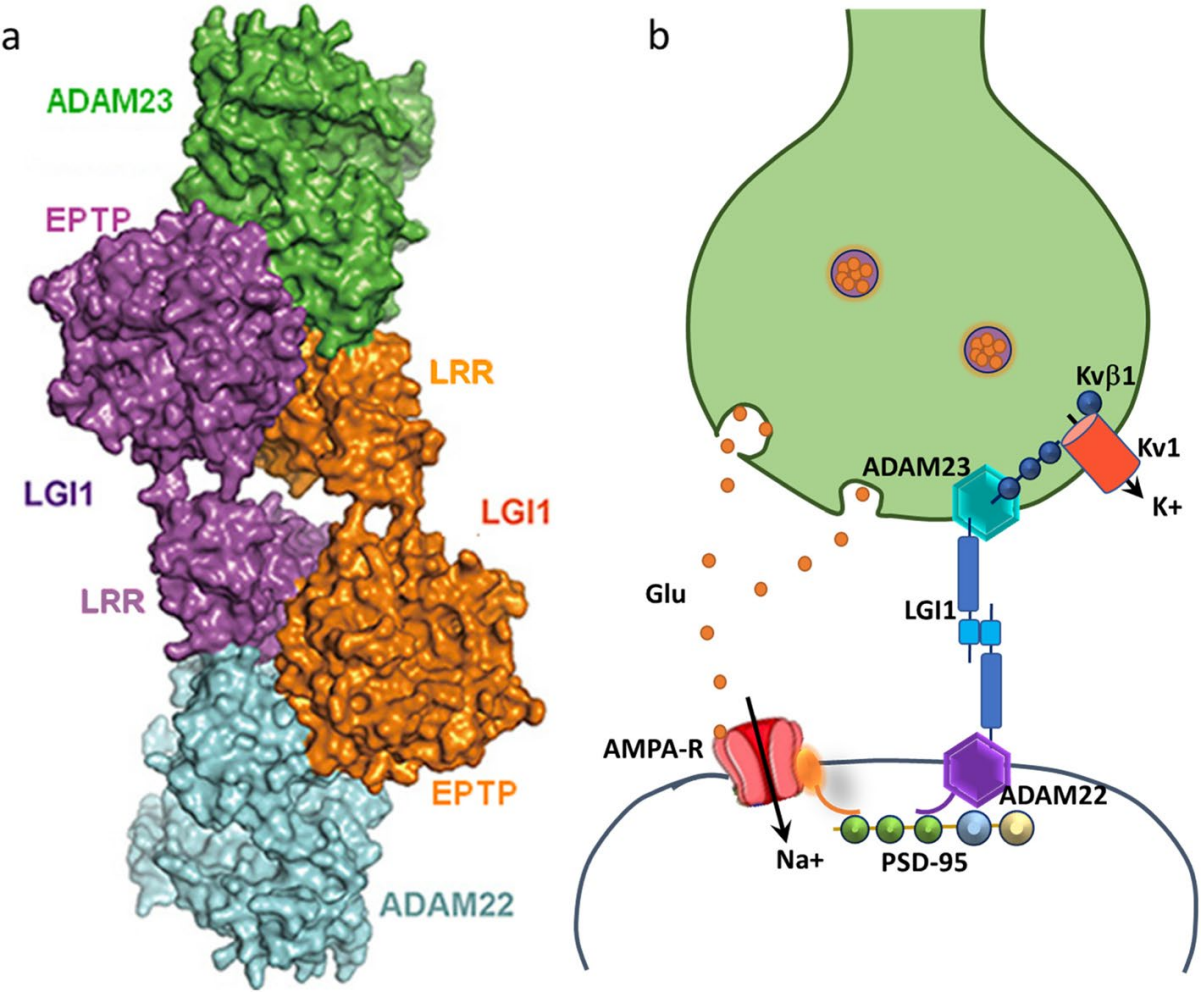
LGI1 protein structure (**a**) and LGI1 interaction with other synaptic proteins (**b**). LGI1: leucin-rich glioma-inactivated 1; LRR: leucin-rich repeat; EPTP: epitempin-repeat; ADAM: a disintegrin and metalloproteinase; Glu: glutamate; Kv: voltage-gated potassium channel; AMPA-R: α-amino-3-hydroxy-5-menthyl-4-isoxazolepropionic acid receptor; PSD-95: postsynaptic density protein 95

LGI1 is a secreted neuronal protein that is primarily expressed in CA1 and CA3 of the hippocampus as well as the dentate gyrus. The protein has two domains: the N-terminal leucine-rich repeat (LRR) domain, which forms LGI1 dimerization, and the C-terminal epitempin-repeat (EPTP) domain, which mediates LGI1 interaction with a disintegrin and metalloproteinase 23/22 (ADAM23/22), a disintegrin, and metalloproteinase (Fig. 3a). The trans-synaptic LGI1-ADAM complex is vital for synaptic transmission and neuronal excitability [18]. At the presynaptic terminal, ADAM23 interacts with the Kv1 subunit of the voltage-gated potassium channel (VGKC), which is essential for the localization of the Kv1.1 and Kv1.2 subunit complexes to the synaptic terminals. At the postsynaptic terminal, ADAM22 interacts with α-amino-3-hydroxy-5-methyl-4-isoxazolepropionic acid receptors (AMPARs) via postsynaptic density protein 95 (PSD95, Fig. 3b) [18]. The exact mechanisms of the effects of autoantibodies against LGI1 are unknown. Nevertheless, research shows that the interaction between the presynaptic LGI1-ADAM23 heterodimer and the postsynaptic LGI1-ADAM22 heterodimer is responsible for synaptic transmission and necessary for hippocampal long-term synaptic plasticity (Fig. 3b). The anti-LGI1 antibody derived from patients’ serum inhibits LGI1 binding to ADAM22 and ADAM23 in vitro, causing a significant reduction in Kv1.1 and synaptic density in the hippocampus of mice, and reversibly reducing synaptic AMPARs in vitro [19]. This provides a potential molecular mechanism for cognitive impairment seen in patients with anti-LGI1 AE. Fels et al. showed that neutralization of LGI1 by anti-LGI1 antibodies reduces AMPAR expression at the surface of both excitatory and inhibitory neurons [19]. They also showed evidence of increased hyperexcitability in mouse hippocampal slices treated with patient’s anti-LGI1 auto-antibodies. Such increased hyperexcitability of the neuronal network is independent from Kv1.1 channels, but rather due to a disturbance of the inhibitory network. Downregulation of the inhibitory network may be a mechanism for the generation of epileptic seizures in patients with anti-LGI1 AE [19].

Clinicians and researchers have observed a variability in the clinical features of anti-LGI1 AE and the lingering sequelae after treatment. Studies have investigated whether epitope specificity and the antibody’s ability to crosslink LGI1 play a crucial role in the pathogenic mechanisms [20–22]. Ramberger et al. found that the administration of the antibody that specifically binds to the LRR domain impairs recognition memory in mice and substantially abrogates long-term potentiation (LTP) induction at CA3-CA1 synapses [20]. Similarly, Extremet et al. discovered that the treatment of neurons with LRR domain-specific antibody increases the intrinsic excitability and reduces the sensitivity to a selective Kv1.1-channel blocker, while the EPTP domain-specific antibody does not [23]. In vitro studies have also found that antibodies binding to the N-terminal LRR domain tend to generate more severe clinical presentations, including FBDS plus seizures, FBDS, and cognitive impairment, whereas those binding to the C-terminal EPTP domain tend to have milder clinical presentations, often with isolated FBDS and absence of cognitive impairment [21]. Ludewig applied purified patient antibodies to mouse hippocampal slices and found that incubation with anti-LGI1 antibodies derived from patients with non-FBDS seizures and cognitive impairment resulted in a significant decline in long-term potentiation or short-term plasticity at CA3-CA1 neurons, as well as decreased hippocampal synaptic density. In contrast, applying anti-LGI1 antibodies derived from FBDS-only patients without cognitive symptoms did not render the same effects [24]. These findings suggest that different epitopes targeted by autoantibodies may play a role in determining the severity of clinical symptoms in anti-LGI1 AE. Therefore, accumulating evidence for the heterogeneities in the cellular and molecular pathogenesis as well as the clinical features of anti-LGI1 AE, underscores the need for vigilance from clinicians and timely treatment with appropriate immunotherapies to avoid widespread disruption of cognitive networks and long-term neurocognitive impairments in verbal and visuospatial memory, executive function, and semantic and phonemic fluency.

## Conclusions

Our findings demonstrate a wide spectrum of clinical, electrographic, and radiological features in anti-LGI1 AE, a disease that has varied disease severities and treatment responses. Anti-LGI1 AE should be considered as a disease with several distinct clinical syndromes. As anti-LGI1 AE has similar clinical presentations as viral encephalitis, Hashimoto's encephalopathy, other forms of autoimmune encephalitis, and rapidly progressive dementia (such as Creutzfeldt-Jakob disease), differential diagnosis is important. When patients present new-onset seizures and are resistant to ASM treatments, early referral to a specialized epilepsy program or center, vEEG monitoring, and serial brain imaging are helpful for further diagnosis clarification and treatment guidance. Multidisciplinary approaches may significantly improve the prognosis, potentially improving patients' quality of life.

## Supplementary Information

**Additional file 1.** Supplement Data for case #5 to explore other etiologies for the cerebral edema.

## Data Availability

The datasets during and/or analyzed during the current study are available from the corresponding author on reasonable request.

## Abbreviations

AchR: Acetylcholine receptor
ADAM: A disintegrin and metalloproteinase
AE: Autoimmune encephalitis
AMPA-R: α-amino-3-hydroxy-5-menthyl-4-isoxazolepropionic acid receptor
ASMs: Anti-seizure medications
CLQT: Cognitive linguistic quick test
CNS: Central nervous system
CSF: Cerebrospinal fluid
EEG: Electroencephalography
EPTP: Epitempin-repeat
FBDS: Faciobrachial dystonic seizures
FDG-PET: Fluorodeoxyglucose positron emission tomography
GAD65: Glutamic acid decarboxylase 65-kilodalton isoform
IVIG: Intravenous immune globulin
LGI-1: Leucine-rich glioma-inactivated 1
LRDs: Lateralized rhythmic discharges
LRR: leucine-rich repeat
LTP: Long-term potentiation
MRI: Magnetic resonance imaging
mRS: Modified rankin scale
PLEX: Plasmapheresis
PSD-95: Postsynaptic density protein 95
TPO: Thyroid peroxidase
vEEG: Video electroencephalography
VGKC: Voltage-gated potassium channel
