# Supplementary material for: Heterogeneity of clinical features, EEG and brain imaging findings in anti-leucine-rich glioma-inactivated protein 1 autoimmune encephalitis: a retrospective case series study and review of the literature
Source: Acta Epileptol. 2023 Aug 15;5:21. doi: 10.1186/s42494-023-00132-5 (PMC11960392; doi:10.1186/s42494-023-00132-5)
Supplement: Supplementary file 1 — Additional file 1. Supplement Data for case #5 to explore other etiologies for the cerebral edema. [file 42494_2023_132_MOESM1_ESM.docx]

**Supplement Data for case #5 to explore other etiologies for the cerebral edema**

**Lab Results**

1. **Toxicology screening**

|  | Latest Reference Range & Units | 06/03/2022 |
| --- | --- | --- |
| AMPHET UR SCREEN | NEGATIVE | NEGATIVE |
| BARBITURATES, UR QL | NEGATIVE | NEGATIVE |
| BENZODIAZ UR SCREEN | NEGATIVE | NEGATIVE |
| COCAINE UR QL | NEGATIVE | NEGATIVE |
| METHADONE UR SCREEN | NEGATIVE | NEGATIVE |
| METHAMPHETAMINE, UR QL | NEGATIVE | NEGATIVE |
| OPIATES UR SCREEN | NEGATIVE | T'FOLLOW |
| OXYCODONE, UR QL SCRN | NEGATIVE | NEGATIVE |
| PCP UR SCREEN | NEGATIVE | NEGATIVE |
| THC UR SCREEN | NEGATIVE | NEGATIVE |
| COMMENT, DRUG PANEL |  | SEE NOTE |
| 6-MONOACETYLMORPHINE, CONFIRMATION, URINE | UNDETECT | UNDETECT |
| CODEINE, CONFIRMATION, URINE | UNDETECT | UNDETECT |
| HYDROCODONE, CONFIRMATION, URINE | UNDETECT | UNDETECT |
| HYDROMORPHONE, CONFIRMATION, URINE | UNDETECT | UNDETECT |
| MORPHINE, CONFIRMATION, URINE | UNDETECT | UNDETECT |
| OXYCODONE, CONFIRMATION, URINE | UNDETECT | UNDETECT |
| OXYMORPHONE, CONFIRMATION, URINE | UNDETECT | UNDETECT |

1. **Malignancy marker**

|  | Latest Reference Range & Units | 06/15/2022 |
| --- | --- | --- |
| CEA | 0.0 - 5.0 ng/mL | 3.9 |
| CA 19-9 | <=37 U/mL | 31 |

1. **CSF studies**

|  | 06/09/23 15:50 |
| --- | --- |
| GRAM STAIN | Rare White Blood Cells; No organisms seen |
| CSF CULT | No growth for 72 hours |
| FUNGUS CULT | No fungus isolated at 3 weeks |
| CRYPTOCOCCUS SP AG EIA | Cryptococcal Antigen negative |
| AFB Stain | No Acid Fast Bacilli seen on smear. |
| AFB CULT | No Acid Fast Bacillus isolated at 3 weeks. |
| EV RNA CSF PCR | NEGATIVE |
| HSV-1 DNA QL, PCR | NEGATIVE |
| HSV 2 DNA, PCR | NEGATIVE |
| WEST NILES VIRUS, PCR | NEGATIVE |

FLOW CYTOMETRY DIAGNOSIS
CEREBROSPINAL FLUID, FLOW CYTOMETRY:  NO IMMUNOPHENOTYPIC ABNORMALITIES DETECTED

CSF Cytology Report:    
FINAL PATHOLOGIC DIAGNOSIS
A:  CEREBROSPINAL FLUID - NEGATIVE FOR MALIGNANCY

**Other imaging study results**

1. **CT angiography head & neck**

| CTA HEAD: Atherosclerotic calcifications of the cavernous internal carotid arteries bilaterally. Mild stenosis in the P1/P2 junction of the left posterior cerebral artery. Vessels are patent. Ectatic basilar artery measuring 5 mm. No aneurysm identified. |
| --- |
|  |
| CTA NECK |
| GREAT VESSELS: Visualized segments are patent. Aortic arch atherosclerosis. |
|  |
| RIGHT ICA: No stenosis or significant plaque. No dissection. |
|  |
| LEFT ICA: No stenosis or significant plaque. No dissection. |
|  |
| VERTEBRAL ARTERIES: Patent extracranial segments. No dissection. |
|  |
| OTHER: Visualized lung apices are clear. No neck mass or suspicious lymph nodes. |

1. **CT chest/Abd**

| CHEST |
| --- |
| HEART AND GREAT VESSELS: Heart size normal. |
| LYMPH NODES: No suspicious lymph nodes. |
| OTHER MEDIASTINUM: No significant abnormality. |
| LUNGS: No suspicious pulmonary nodules. No parenchymal consolidations. |
| PLEURAL SPACE: Normal. |
| SOFT TISSUES: No significant abnormality. |
|  |
|  |
| ABDOMEN/PELVIS |
| LIVER: Within normal limits for technique. |
| GALLBLADDER/BILIARY TREE: No calcified gallstones. No biliary dilation. |
| SPLEEN: Within normal limits for technique. |
| PANCREAS: Within normal limits for technique.  No suspicious pancreatic abnormality. |
| ADRENALS: No adrenal nodules. |
| KIDNEYS: No significant abnormality. |
| GI TRACT: No significant abnormality. |
| MESENTERY/LYMPH NODES:  No suspicious lymph nodes. |
| PERITONEUM: No free air or fluid. |
| AORTA/VESSELS: Normal abdominal aortic diameter (<3cm). |
| BLADDER: No significant abnormality. |
| PELVIC STRUCTURES: No significant abnormality. |
| ABDOMINAL WALL/SOFT TISSUES: No significant abnormality. |
|  |
|  |
| BONES: |
| There are no destructive osseous lesions identified. |

1. **Body PET scan**

| No specific FDG-avid sign of active malignancy to suggest paraneoplastic syndrome. Incidental survey PET/CT findings as follows: |
| --- |
|  |
| 1. Increased metabolic conspicuity in the gastric pyloric region is nonspecific. Consider EGD for further evaluation. Favor inflammatory/reactive uptake at the GE junction. |
|  |
| 2. Incidental focal uptake in the sigmoid colon. Though possibly inflammatory. |
|  |
| 3. Low-grade uptake in small right hilar, and right perihilar lymph nodes, possibly inflammatory/reactive. |
|  |
| 4. Probable dental carry in a right mandibular tooth with a right level Ib reactive lymph node. Mild asymmetric uptake in the right base of tongue is nonspecific. Consideration could be given to direct visualization. |
